# Women’s approval of domestic physical violence against wives: analysis of the Ghana demographic and health survey

**DOI:** 10.1186/s12905-015-0276-0

**Published:** 2015-12-21

**Authors:** David Teye Doku, Kwaku Oppong Asante

**Affiliations:** Department of Population and Health, University of Cape Coast, Private Mail Bag, University Post Office, Cape Coast, Ghana; University of KwaZulu-Natal, Durban, South Africa

**Keywords:** Justification of physical domestic violence, Socio-demographic factors, Women’s health, Intimate partner violence

## Abstract

**Background:**

Intimate partner violence (IPV) has serious consequences for the physical, psychological, and reproductive and sexual health of women. However, the factors that make women to justify domestic violence against wives in many sub-Saharan African countries have not been explored. This study investigates factors that influence women approval of domestic physical violence among Ghanaian women aged 15–49.

**Method:**

A nationally representative sampled data (*N* = 10,607) collected in the 2003 and 2008 Ghana Demographic and Health Survey were used. Multivariate logistic regression was used to study the associations between women’s economic and socio-demographic characteristics and their approval of domestic physical violence against wives.

**Results:**

Women aged 25–34 and 15–24 were 1.5 and 1.3 times, respectively, more likely to approve domestic physical violence against wives compared to those aged 35 years and above. Furthermore, women with no education (OR = 3.1, CI = 2.4–3.9), primary education (OR = 2.6, CI = 2.1–3.3) and junior secondary education (OR = 1.8, CI = 1.4–2.2) had higher probability of approving domestic physical violence compared to a woman who had secondary education or higher. Compared to women with Christian belief, Moslems (OR = 1.5, CI = 1.3–1.8) and Traditional believer (OR = 1.7, CI = 1.2–2.4) were more likely to approve domestic physical violence of wives. Women who were in the richest, rich and middle wealth index categories were less likely to approve domestic physical violence of wives compared to the poorest.

**Conclusion:**

These findings fill a gap in understanding economic and socio-demographic factors associated with approval of domestic physical violence of wives. Interventions and policies should be geared at contextualizing intimate partner violence in terms of the justification of this behaviour, as this can play an important role in perpetration and victimization.

## Background

Intimate partner violence (IPV), defined as actual or threatened physical, sexual, psychological and emotional abuse by current or former partners, is currently a public health concern globally. According to World Health Organization, the global prevalence of physical and/or sexual intimate partner violence women was 30 % [1] and the prevalence is very high in countries from sub-Saharan Africa and South-East Asia [2]. Intimate partner violence is known to be associated with both short and long term psychological and mental health problems including depression, anxiety, tendencies towards addiction and suicidal ideation [3–5]. In Ghana, the prevalence of IPV has been reported to be low-fewer than 4 % of women report ever experiencing any victimization [6]. Reasons attributed to the low reported cases of such violence was that such cases of IPV may be considered less appropriate, not worthy of people’s attention and sometimes dependent on the nature of physical violence their husbands use.

Gender equity is an important step in women empowerment. In many societies, norms and cultural practices justify that men have the right to use force against women [7, 8]. These practices have serious consequences for the physical, psychological, and reproductive and sexual health of women [9, 10]. Approval of partner violence and its psychosocial effects have been documented [11, 12]. Most of these studies showed that the rate of justification and approval of domestic physical violence against wives in many countries are quite high and can vary by the reason for abuse (e.g. infidelity, neglect of child). Furthermore, these studies found that women tended to approve of IPV (including physical violence) at a greater rate than men, and factors reflecting lower socio-economic status is associated with typically higher acceptance of IPV [8].

One of the reasons for the acceptance of violence against women, especially in sub-Saharan Africa and South-East Asia countries has been attributed to patriarchy [13, 14]. Patriarchy reflects social attitudes and norms around the role of women in relation to men as a source of partner violence [14]. The power of male superiority as reflected in studies showed that large percentages of both men and women believe that male violence against women are acceptable under different circumstances [7, 8, 15]. This ideology thus plays an important role acceptance and or approval of IPV towards women, where the behaviours of women are construed to be the triggers of violence by the partners [13], and women generally accepts this power exertion on them [16].

There is evidence of the existence of patriarchy in Ghana [17, 18], as men are considered wise, strong and placed in positions of authority compared to women. Male dominance has also resulted in low literacy rate of 63.5 % compared to the male rate of 78.3 % [19]. A study among university students from Ghana showed that the frequency of use of controlling behaviours and victimization/perpetration amongst men and women (e.g. control the other’s money) were similar, and controlling behaviours were seen to be associated with IPV [20]. A recent study conducted in Ghana has suggested that women who reported physical, psychological and sexual violence were more likely to have suicidal thoughts, sleep deprivation and fear of partners or husbands [21].

Within this context, it is further relevant to understand the role of socio-demographics such as age, place of residence, marital status, education, occupational status and wealth in the dynamics of domestic physical violence against wives across the globe [7]. We focus on Ghana because some previous studies have reported that less than 5 % of all women have reported being abused in one form or another [22]. Despite this observation, there is a paucity of scholarly research on women’s approval of domestic physical violence against women by their husbands and or partners especially using data at the national level. The aim of this study is to investigate factors that influence women approval of domestic physical violence against women in Ghana using data from the 2003 to 2008 Ghana Demographic and Health Survey.

### Theoretical framework

The resource theory and the subculture-of-violence theory were used to explain intimate partner violence (IPV) and its approval [23–25]. Resource theory indicates that the availability of resources to both men and women, determines the nature and magnitude of violence among partners. Some researchers [23, 24] have argued that the imbalance in IPV among income groups occurs as a result of the fact that individuals with lower socio-economic status (i.e. income and social status) may have fewer legitimate resources to utilize to attain power. Some proponents of the resource theory have suggested that the availability of resources for women in particular, may to some extent alter the dependency relationship between men and women, and possibly lower men’s dominance over women in the domestic space. Applying the resource theory to women’s approval of domestic physical violence against women, we can argue that women’s financial independence and autonomy provides some form of protection against physical violence. The lack of such resources may not only make women vulnerable to IPV, but also to the approval of such violence act. Thus, lower levels of SES (i.e. income, education and occupation) may predispose women to accepting violence against women.

The subculture-of-violence theory developed by Wolfgang and Ferracuti [25] posits that the occurrence of violence is not evenly distributed among groups in the social structure; it is concentrated in poor urban areas. According to these theorists, since violence is known to occur frequently among a specific subset of the larger community, it is believed that, there is a value system at work in that subculture that makes violence more likely. According to Wolfgang and Ferracuti [25], “there is a potent theme of violence present in the cluster of values that make up the life-style, the socialization process, the interpersonal relationships of individuals living in similar conditions”(p. 140). This suggests that individuals found in that sub-culture learn the values and norms of violence through socialization and social control in their environment. In other words, violence is learned socially and passed on by group members, thus sustaining the subculture of violence. In applying the subculture-of-violence theory to this study, we hypothesized that certain subcultures of society, measured by the socio-demographic characteristics relate to justification of intimate partner violence, particularly physical domestic violence against wives.

## Methods

### Data

We utilized a national representative data (*N* = 10,607) from the Ghana Demographic and Health Surveys (GDHS) conducted in 2003 and 2008. The Ghana Demographic Health Survey is a nationwide survey with a representative sample of women and men aged 15–49 and 15–59, respectively. The present study used the women questionnaire in 2003 (*N* = 5691) and 2008 (*N* = 4916). All the surveys used a two stage sample based on the Ghana Population and Housing Census to produce separate estimates for key indicators for each of the ten regions in Ghana. The first stage involved selecting sample points or clusters from an updated master sampling frame constructed from the Ghana Population and Housing Census. The second stage of selection involved systematic sampling of 30 of the households listed in each cluster. This was done to ensure adequate numbers of completed individual interviews to provide estimates for key indicators with acceptable precision and to provide a sample large enough to identify adequate numbers of under-five deaths to provide data on causes of death. The clusters were selected using systematic sampling with probability proportional to size. Each household selected for the GDHS was eligible for interview with the household questionnaire. In half of the households selected for the survey, all eligible women aged 15–49-year-old were interviewed with the women’s questionnaire. In 2008, data was not administered in one cluster due to security concerns. The data collection took place over a three-month period, from early September to late November. The response rates were generally very high for example, 93.8 and 95.8 % in  2003 and 2008, respectively. The main reason for non-response was the failure to find individuals at home despite repeated visits to their household. Ethical approval for the study protocol was given by the Ghana Health Service Ethical Review Committee in Accra, Ghana.

### Dependent variable

Respondents were asked whether a husband is justified in beating his wife under a series of circumstances, that is if: wife burns the food; wife argues with him, wife goes out without telling him, wife neglects the children, and wife refuses to have sex with him. The response format to these questions were yes = 1 and no = 0.

### Independent variables

A number of independent variables were used based on previous studies [1–5, 7]. The independent variables used in this study included place of residence (urban and rural) and age categorized as 15–19, 20–24, 25–29, 30–34, 35–39, 40–44 and 45–49 years. To increase enough power for the study age was re-categorized as 15–24, 25–34 and 35–49 years for the logistic regression analysis. The rest of the independent variables were marital status (never married, currently married and formerly married), religion (Christian, Traditional, Moslem and Others) and household wealth, represented by wealth index (in five categories from poorest to richest). The wealth index was constructed using data on a household’s ownership of selected assets, such as televisions and bicycles; materials used for housing construction and types of water access and sanitation facilities. This constructing of wealth index using these items have previously been used in Ghana [26, 27]. The index places individual households on a continuous scale of relative wealth. It was then categorized into five (poorest, poorer, middle, richer, and richest). In addition, education (coded as; no education, primary, secondary and higher) was used.

### Statistical analysis

First, Pearson Chi-square test was used to examine the relationship between socio-demographic factors and the indices of attitudes towards domestic violence against wives by husband. Next, univariate and multivariate logistic regression analyses were conducted to assess the association between women’s socio-demographic characteristics and attitude towards domestic violence against wives by husbands. The results from the logistic regression analyses are presented as odds ratios (OR) with 95 % confidence intervals (CIs). Statistical significance was defined as a two-tailed *p* value < 0.05 in all analyses. The SPSS statistical package (version 21) was used to conduct data analyses.

## Results

### Sociodemographic characteristics of participants

The socio-demographic characteristics of the participants are presented in Table 1. Approximately 39, 30 and 31 % of the women were in the age groups of 15–24, 25–34 and 35–49 years, respectively. The majority (75.7 %) were working and currently married (62.6 %), and Christians (73.5 %). The results as presented in Table 1 further shows that over half (57.2 %) of the participants lived in the rural areas, and about 22.90 and 21.4 % were found in the wealth index of poorest and richest, respectively. The majority (67.1 %) of the participants had primary or junior secondary school as the highest level of educational qualification.

**Table 1:** Sociodemographic characteristics of participants

Characteristics		Number	Percent
Age groups (*N* = 4917)			
	15-24	1903	38.8
	25-34	1478	30.2
	35-49	1536	31.0
Level of education (*N* = 10603)			
	No education	3160	29.8
	Primary	2111	19.9
	Secondary	5007	47.2
	Higher	325	3.1
Occupation (*N* = 10,566)			
	Working	7998	75.7
	Not working	2568	24.3
Marital status (*N* = 10,607)			
	Never married	3055	28.8
	Currently married	6644	62.6
	Formerly married	908	8.6
Place of residence (*N* = 10,607)			
	Urban	4536	42.8
	Rural	6071	57.2
	Religion (*N* = 10,603)		
	Christian	7794	73.5
	Traditional	476	4.5
	Moslem	1845	17.4
	Others	488	4.6
Wealth Index (*N* = 10,607)			
	Poorest	2428	22.9
	Poor	1920	18.1
	Middle	1887	17.8
	Rich	2104	19.8
	Richest	2268	21.4

*N* = Number; % = Percentage of *N*

### Socio-demographic factors and attitude towards physical domestic violence of wives

The prevalence of the approving at least one form of domestic violence against wives was 39 %. The relationship between socio-demographic factors and attitudes towards domestic violence are presented in Table 2. Age, level of educational, marital status, place of residence, religion and wealth index were related to all the indices of attitudes towards domestic physical violence. Occupational status was related to only three indices of domestic physical violence namely going out without telling husband, refusal to have sexual intercourse with husband and burning of food. All the background variables in this study, with the exception of occupational and marital status, were related to women agreeing to at least one measure of attitudes towards domestic violence (Table 2).

**Table 2:** Attitude towards domestic physical violence by background characteristics among Ghanaian women age 15–49 year old 2003-2008

Variable		Goes out without telling him	Neglects the children	Argues with him	Refusal to have sexual intercourse with him	Burns the food	At least one reason
Age							
	15-24	23.8***	27.4***	22.4***	10.7***	9.8***	39.4**
	25-34	22.4	27.1	21.1	13.2	8.0	35.9
	35-49	30.8	24.4	20.3	13.3	7.4	34.4
Level of education							
	No education	32.9***	34.7***	30.7***	21.2***	12.1***	49.9***
	Primary	24.9	31.3	24.5	14.4	10.4	42.4
	Secondary	18.3	22.5	17.7	8.5	6.5	31.6
	Higher	6.3		6.3	5.2	2.6	11.1
Occupation							
	Working	22.4***	26.6	21.4	12.8***	7.9*	36.7
	Not working	22.1	25.4	20.8	10.4	9.5	36.7
Marital status							
	Never married	20.4***	24.0**	20.4***	8.7***	7.5***	34.8
	Currently married	23.3	27.7	22.0	13.7	9.0	37.6
	Formerly married	22.2	26.0	20.3	15.4	6.9	37.8
Place of residence							
	Urban	16.1***	20.4***	16.0***	8.8***	5.5***	28.7***
	Rural	28.0	31.8	26.3	15.5	10.9	44.4
Religion							
	Christian	19.6***	24.3***	19.3***	10.2***	7.2***	33.4***
	Traditional	35.0	39.8	35.4	25.9	15.6	56.0
	Moslem	31.6	31.5	26.5	18.6	10.4	48.1
	Others	26.2	32.5	28.8	16.8	11.9	42.5
Wealth Index							
	Poorest	33.5***	35.5***	33.0***	21.3***	12.9***	54.1***
	Poor	30.2	32.9	27.4	16.2	11.8	45.0
	Middle	23.4	28.3	21.3	10.8	8.3	37.9
	Rich	17.8	23.0	18.3	10.1	6.7	32.3
	Richest	11.6	16.3	11.6	6.2	4.0	22.5

*p < 0.05; **p < 0.01; ***p < 0.001

**Table 3:** Odds ratios (OR) and their 95 % confidence intervals (CI) for factors associated with approval of domestic violence against wives^a^

Predictors		Agrees with at least one form of violence Model I^b^ OR (95 % CI)	Agrees with at least one form of violence Model II^c^ OR (95 % CI)
Age			
	35-49	1.0	1.0
	25-34	1.14 (1.10-1.25)*	1.47 (1.20-1.76)*
	15-24	1.10 (1.02-1.24)*	1.30 (1.13-1.49)*
Level of education			
	Higher	1.0	1.0
	Secondary	2.10 (1.72-2.56)**	1.77 (1.44-2.18)*
	Primary	3.67 (2.95-4.55)**	2.59 (2.05-3.27) **
	No education	5.66 (4.62-6.95)***	3.10 (2.43-3.94)***
Occupation			
	Working	1.0	1.0
	Not working	0.93 (0.85-1.02)	1.07 (0.91-1.25)
Marital status			
	Never married	1.0	1.0
	Currently married	1.33 (1.22-1.45)*	1.11 (0.93-1.33)
	Formerly married	1.04 (0.89-1.21)	0.99 (0.77-1.28)
Place of residence			
	Urban	1.0	1.0
	Rural	2.00 (1.85-2.17)*	1.19 (1.01-1.42)*
Religion			
	Christian	1.0	1.0
	Moslem	2.06 (1.85-2.29)**	1.53 (1.31-1.80)*
	Traditional	2.87 (2.24-3.52)***	1.69 (1.19-2.38)**
	Others	1.86 (1.50-2.40)*	1.04 (0.80-1.35)
Wealth Index			
	Richest	1.0	1.0
	Rich	1.76 (1.55-2.00)*	1.64 (1.37-1.96)*
	Middle	2.16 (1.89-2.46)**	1.91 (1.55-2.35)*
	Poor	2.80 (2.46-3.18)**	2.25 (1.78-2.85)**
	Poorer	4.74 (4.18-5.38)***	3.48 (2.73-4.44)***

^a^Computed from the 2003 and 2008 Ghana Demographic and Health Survey

^b^Model I = Univariate model

^c^Model II = Multivariate model of all independent variable statistically significant at the bivariate level

*p < 0.05; **p < 0.01; ***p < 0.001

A similar pattern was observed in Model II, albeit with reduced magnitude, women with no education were about 3.1 times (OR = 3.1, CI = 2.4–3.9) more likely to approve physical domestic violence of wives. Women with primary education (OR = 2.6, CI = 2.1–3.3) and junior secondary education (OR = 1.8, CI = 1.4–2.2) were 2.6 times and 1.8 times, respectively, more likely to approve physical domestic violence against wife than women with secondary education or higher. The results in Model I, further suggests that currently married women were 1.3 times (OR = 1.3, CI = 1.2–1.45) more likely to approve physical domestic violence of wives than those who were never married. Rural residency (OR = 1.2, CI = 1.0–1.2) increased the probability of a woman approving physical domestic violence of wife compared to urban residency.

The results from Table 3 further indicate that in Model I as compared to Christian women, Moslem (OR = 2.1, CI = 1.9–2.3), traditional believers (OR = 2.9, CI = 2.2–3.5) and women of other faith (OR = 1.9, CI = 1.5–2.4) were more likely to approve physical domestic violence. In Model II these associations remained although they attenuated slightly, and the relationship between approval of physical violence against wives and other faith lost its statistical significance. Furthermore, the result shows that the richer a woman the less likely that she would approve physical violence against wives. Women who were in the poorer (OR = 3.5, CI = 2.7–4.4), poor (OR = 2.3, CI = 1.8–2.9), middle (OR = 1.9, CI = 1.6–2.4) and rich (OR = 1.6, CI = 1.4–2.0) quintiles on the wealth index were more likely to approve physical domestic violence against wives compared to their counterparts who were in the richest category.

## Discussion

This study was conducted to examine socio-demographic trends in women’s approval of domestic physical violence among Ghanaians women using data from 2003 to 2008 Ghana Demographic and Health Survey. This study found that several socio-demographic factors were associated with the likelihood of a woman approving domestic physical violence against wives. Age, level of education, place of residence, religion and wealth index were independently associated with approval of physical violence against wives. The younger a woman, the more likely that she would approve domestic violence against wives. No consistent trend was found between marital status and approval of physical domestic violence against wives. The relationship between level of education and women’s approval of domestic physical violence against wives were mainly negative and significant such that women with lower education had higher chances of approving domestic physical violence against wife compared to those with higher education. Furthermore, women of the Moslem and Traditional beliefs were both more likely to approve physical domestic violence against wife than those with Christian beliefs. The findings also showed that women who were in the richest, rich and middle wealth index categories were less likely to approve physical domestic violence against wife compared to those in the lower wealth category.

In this study, women aged 25–34 and 15–24 had higher probability of approving domestic violence against wives compared to those aged 35 years and above. This therefore suggests that the younger a woman, the likelihood of accepting domestic violence against wives. A woman’s age has been shown to affect the perception and attitudes towards physical domestic violence against wives [8, 28–30]. There is, however, no consistency in the literature. While some studies have found that age decrease the likelihood of approving physical violence against women [8] other studies indicated otherwise [28, 29]. In some other studies, approving of domestic violence of women is dependent on the type of offence committed. For example, Waltermaurer et al. [8] found out that Georgian woman less than 25 years of age were more likely to justify physical domestic violence against wives when a woman refuses sex compared to women aged 25–34. The same study further indicated that older women were more likely to justify physical domestic violence against wives around issues of infidelity or going out without permission compared to the middle age group [8]. A recent study in Ghana indicated that the age difference between younger women and their older male partners can create a “father-daughter” relationship, leading to emotional violence [31]. In the same vein, this relationship could lead to the acceptance or approval of physical domestic violence against wives as found in this study.

We found that the lower a woman’s education, the more likely that she would accept domestic physical violence against women. We found that women with no education, primary education and junior secondary education had higher probability of approving physical domestic violence against wives compared to a woman who had secondary education or higher. Furthermore, a gradient exist in these relationships, implying that the higher a woman’s education, the lower the likelihood of approving physical domestic violence against wife. Similar findings had been reported in a recent study conducted in Malawi [32], and an extensive literature review on women’s attitudes towards physical domestic violence against wives in 25 countries in Sub-Saharan African countries [33]. Similarly, Mann and Takyi [15] reported that Ghanaian women with no education were more likely to support violent ideologies, suggesting that higher education could lead to the reduction in supporting violent ideologies. Education does not only provide important information for decision making but also encourages empowerment and autonomy. These numerous benefits of education is vital in influencing women’s perception about domestic violence. The inverse relationship between education and acceptance of domestic violence of wives among women could be attributed to the fact that educated women perceive domestic violence as a negative phenomenon which could have physical and psychological harm on the victim [15], while less educated women are less informed about the consequences of such behaviour.

Consistent with previous studies in Kenya [34], Uganda [35] and elsewhere [36], rural residency was found to increase the likelihood of accepting domestic physical violence. Disparities in women empowerment campaigns, especially to those living in rural setting, as well as the lack of exposure to new forms of interpersonal relationship management including domestic violence could account for the rural–urban differences in justification of domestic physical violence.

Traditional faith practitioners had the likelihood to approve physical domestic violence of wife compared to Christians. Also, Moslems were more likely to approve physical domestic violence against wives compared to Christians. A study conducted among Egyptian, Palestine, Israelis and Tunisian women found that religion (Islamic) was associated with acceptance of violence against wives, as selective excerpts from the Koran are erroneously used to justify it [37]. Religion which exercises a strong regulatory system may have different shared social values among its members. This could therefore confirm the findings in the present study regarding differences among women in approving domestic physical violence of wives among women by religion. It would be important for religious leaders especially those in Africa to educate their members about the physical and psychological effect of domestic violence against women.

Women who were in the richest, rich and middle wealth index categories were less likely to approve physical domestic violence wives compared to those from lower wealth index. A previous study in Ghana has found a similar result where a significant relationship was found between economic dependency and IPV [15]. The mechanism underlying the relationship between wealth index and approval of physical domestic violence of wives by women could be explained from the resource theory, along with women financial independence, where there is less today reliance on men for their source of living. This finding suggests that improving the standards of living of women could have an effect on women’s perception of domestic violence against wives.

The findings of this study must be interpreted cautiously in the light of some important limitations. This study is limited by its cross-sectional nature and hence causal inferences cannot be made. Furthermore, the study relied on self-report measures, which could be affected by social desirability bias or memory bias. Future research is also needed to ascertain which social as well as cultural factors are influencing women’s justification of domestic violence again wives. Despite these shortcomings, the study has compelling strengths. First, the large sample size gave the study sufficient power. Additionally, the representativeness of the sampling strategy as well as the nationwide nature of the data boosts the study’s generalizability to other settings.

## Conclusions

In summary, age, level of education, place of residence, religion and wealth index were found to be associated with Ghanaian women’s approval of domestic physical violence against women. This study contributes to the scanty literature on the influence of socio-demographic factors in relations to women’s attitude towards domestic violence against women in countries in sub-Saharan Africa and Ghana in particular. Interventions and policies should be geared at contextualising intimate partner violence in terms of the approval of this behaviour, as this can play an important role in perpetration and victimization. These interventions must target women with low educational status and less wealth.
